# The methodological quality of systematic reviews comparing temporomandibular joint disorder surgical and non-surgical treatment

**DOI:** 10.1186/1472-6831-8-27

**Published:** 2008-09-26

**Authors:** Ricardo V Bessa-Nogueira, Belmiro CE Vasconcelos, Richard Niederman

**Affiliations:** 1 University of Pernambuco, School of Dentistry, Recife, Brazil; 2 Forsyth Institute, Boston, Massachusetts, USA; 3 Boston University, Goldman School of Dental Medicine, Boston, Massachusetts, USA

## Abstract

**Background:**

Temporomandibular joint disorders (TMJD) are multifactor, complex clinical problems affecting approximately 60–70% of the general population, with considerable controversy about the most effective treatment. For example, reports claim success rates of 70% and 83% for non-surgical and surgical treatment, whereas other reports claim success rates of 40% to 70% for self-improvement without treatment. Therefore, the purpose of this study was to (1) identify systematic reviews comparing temporomandibular joint disorder surgical and non-surgical treatment, (2) evaluate their methodological quality, and (3) evaluate the evidence grade within the systematic reviews.

**Methods:**

A search strategy was developed and implemented for MEDLINE, Cochrane Library, LILACS, and Brazilian Dentistry Bibliography databases. Inclusion criteria were: systematic reviews (± meta-analysis) comparing surgical and non-surgical TMJD treatment, published in English, Spanish, Portuguese, Italian, or German between the years 1966 and 2007(up to July). Exclusion criteria were: *in vitro *or animal studies; narrative reviews or editorials or editorial letters; and articles published in other languages. Two investigators independently selected and evaluated systematic reviews. Three different instruments (AMSTAR, OQAQ and CASP) were used to evaluate methodological quality, and the results averaged. The GRADE instrument was used to evaluate the evidence grade within the reviews.

**Results:**

The search strategy identified 211 reports; of which 2 were systematic reviews meeting inclusion criteria. The first review met 23.5 ± 6.0% and the second met 77.5 ± 12.8% of the methodological quality criteria (mean ± sd). In these systematic reviews between 9 and 15% of the trials were graded as high quality, and 2 and 8% of the total number of patients were involved in these studies.

**Conclusion:**

The results indicate that in spite of the widespread impact of TMJD, and the multitude of potential interventions, clinicians have expended sparse attention to systematically implementing clinical trial methodology that would improve validity and reliability of outcome measures. With some 20 years of knowledge of evidence-based healthcare, the meager attention to these issues begins to raise ethical issues about TMJD trial conduct and clinical care.

## Background

Temporomandibular Joint Disorders (TMJD) is a collective term used to describe a number signs and symptoms involving the temporomandibular joints, masticatory muscles, and associated structures. Approximately 60–70% of the general population has at least one sign of a temporomandibular disorder which include limited mouth opening, clicking, and locking (e.g.: [1,2]). Further, TMJD is frequently associated with pain in regions outside of the immediate joint area, such as recurrent headaches and neck pain[1,3]. Patients afflicted with a severe TMJD can experience significant reductions in quality of life, affecting both personal life and work, and everyday activities such as eating, talking, yawning, and laughing can become painful [1-3].

The treatment of TMJD can be divided into two main groups. The first is the non-surgical therapy and it includes treatments such as counselling, physiotherapy, pharmacotherapy, and occlusal splint therapy (e.g.:[1,4]). The other is the surgical therapy and it ranges from temporomandibular joint arthrocentesis and arthroscopy to the more complex open joint surgical procedures, referred to as arthrotomy (e.g.:[5]). Narrative reviews indicate that the success rate of nonsurgical treatment is approximately 70% (e.g.:[1]) and the surgical treatment success is approximately 83% (e.g.:[5]), whereas other studies report approximately 40% to 70% self-improvement without any treatments (e.g.:[6,7]). Systematic reviews, however, paint a different picture. The systematic reviews indicate that there is not enough high quality evidence to make informed clinical decisions (e.g.: [8-17]). Yet, some systematic reviews do offer treatment guidance. Clearly, there is some controversy about which treatments are the most effective.

To provide a baseline of high quality information, Bader and Ismail [18] performed a literature survey identifying published systematic reviews of on clinical dentistry, of which 7 examined TMJD. Two addressed the cause and effect of TMJD, one study addressed diagnosis and imaging techniques, and the remaining 4 reviews were about the treatment of TMJD. None addressed TMJD surgical treatment.

The purpose of the current study, therefore, was to (1) identify systematic reviews comparing temporomandibular joint disorder surgical and non-surgical treatment, (2) evaluate their methodological quality, and (3) evaluate the evidence grade within the systematic reviews.

## Methods

A search strategy (Table 1) was developed to identify systematic reviews (SR) that address TMJD treatment. The search comprised articles indexed in MEDLINE, Cochrane Library, LILACS, and Brazilian Dentistry Bibliography databases that were published in English, Spanish, Portuguese, Italian, and German between the years 1966 and 2007 (up to July, 31).

**Table 1:** Search Strategy

*MEDLINE, Cochrane Library, LILACS, and Brazilian Dentistry Bibliography between the years 1966 and 2007 (up to July, 31)*		
Review **OR** Review Literature **OR** Meta-Analysis	**AND**	Temporomandibular Joint/surgery **OR** Temporomandibular Joint Disk/surgery **OR** Craniomandibular Disorders/surgery **OR** Temporomandibular Joint Disorders/surgery **OR** Temporomandibular Joint Dysfunction Syndrome/surgery

The following inclusion criteria were used to identify potentially relevant papers: the article was really a systematic review or meta-analysis, as stated and described in methods section of the publication, and the primary focus was a comparison among different TMJD surgical treatments and non-surgical treatment. Exclusion criteria were: systematic reviews addressing non-surgical TMJD only; clinical trials, narrative reviews or editorials or letters to editor; *in vitro *or animal studies; articles published in languages other than English, Spanish, Portuguese, Italian or German. Initially, two investigators (RVBN and RN) independently read all the titles and abstracts from the multiple search results to identify potentially eligible articles for inclusion. All potentially eligible studies were then retrieved and full-text article was reviewed to determine if it met the inclusion criteria. Disagreements were resolved by discussion among the investigators.

In the next phase, the investigators independently evaluated the identified systematic reviews using three instruments (AMSTAR, OQAQ and CASP) to appraise the methodological quality of systematic review [19-21].

AMSTAR[19] or 'Assessment of Multiple Systematic Reviews' consists of 11 items tool and was developed pragmatically using previously published tools and expert consensus. The original 37 items were reduced to an 11 item instrument addressing key domains (eg. study question, search strategy, inclusion/exclusion criteria, data extraction, study quality). The resulting instrument was judged by the expert panel to have face and content validity[19] and The Canadian Agency for Drugs and Technologies in Health (CADTH) was selected AMSTAR as the best instrument for appraising systematic reviews[22,23].

OQAQ[20] or 'Overview Quality Assessment Questionnaire' has nine individual items and it specified purpose is to evaluate the scientific quality (i.e., adherence to scientific principles) of systematic reviews published in medical literature. This scale is not designed to measure literary quality or importance. The validity of the scale has been tested and validated[20].

The Critical Appraisal Skills Programme or CASP was developed by the Public Health team in Oxford[24] and the programme of North Thames Research Appraisal Group (NTRAG). It aims to enable clinicians to develop the skills to find and make sense of research evidence, helping them to put knowledge into practice. CASP uses an instrument to appraise systematic reviews based on 10 questions[21]. These questions address key domains (eg. comprehensive search, validity assessment, results combination) of methodological quality.

To have a standard way to compare, the answers of each instrument were scored 'yes' or 'no' when the criterion was explicitly met or not met, and 'can't tell' if methods were reported incompletely or not reported at all. For the calculation of the overall methodological quality the mean of the number of 'yes', 'can't tell', 'no' answers recorded for each instrument were determined.

To appraise the quality of the underlying evidence, we used the GRADE system [25,26]. Essentially GRADE classifies quality in four categories: randomized clinical trials (RCT) that show consistent results (high quality); randomized clinical trials with methodological limitations or QUASI-RCT (moderate quality); and observational studies with control groups (e.g., cohort and case-control studies) without exceptional strengths (low quality); unsystematic clinical observations (e.g., case reports and case series) as evidence of very low quality evidence (very low quality).

The number of reports that met each of the criteria was determined and tabulated. The estimated mean overall quality score was calculated using Excel for windows (Microsoft, USA) spreadsheets.

## Results

### Search results

**Figure 1:**
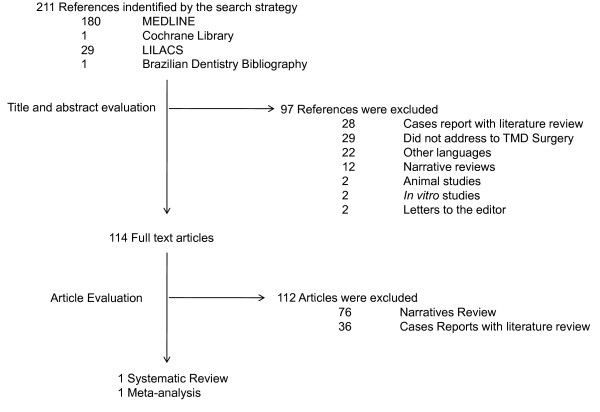
Flow chart showing the results of the search strategy.

Figure 1 summarizes the results of the Medline, Cochrane Library, LiLACS and Brazilian Dentistry Bibliography databases searches. 211 references that appeared to meet the definition of systematic review or meta-analysis were identified (Additional file 1). Inspection of the titles and abstracts by two investigators excluded 97 as not meeting inclusion criteria, leaving 114 that appeared to be relevant or which abstracts were missing. Full text of the 114 references was obtained and their reference lists examined for additional relevant articles. Of the 114, 112 were eliminated leaving 2 articles that met all inclusion criteria.

The included systematic reviews are summarized in Table 2. The first systematic review, Kropmans et al [27] addressed the question: what are the positive effects of arthroscopic surgery, arthrocentesis and physical therapy with regard to TMJD. The second, Reston and Turkelson [28], addressed the question: can any surgical procedures, compared to non-surgical therapy effectively treat TMJD?

**Table 2:** Summary of the included systematic reviews

*Year of Publication*	*Authors*	*Included Surgical Procedures*	*Specific Outcome*		*Conclusion*
1999	Kropmans et al [27]	Arthroscopic surgery Arthrocentesis	All studies claimed effectiveness of the therapeutic intervention(s). 11 (35%) of the studies compared different sets of therapeutic interventions but none of them found a statistical significant difference between the effects of different interventions.		No differences in effects on MMO and pain intensity or mandibular function impairment were found between arthroscopic surgery, arthrocentesis and physical therapy.
2003	Reston and Turkelson [28]	Arthrocentesis Arthroscopy Disc repair/reposition	Arthroscopy vs Arthrocentesis (1)	0.08 (95% CI: – 1.05 to 1.21) p = 0.446548 (2) 0.21 (95% CI: – 0.35 to 0.77) p = 0.230041 (3) 0.22 (95% CI: – 0.36 to 0.79) p = 0.231138 (4)	There were no statistically significant differences in the outcomes of patients given these different treatments regardless of the improvement rate we assumed for the control group.
			Arthroscopy vs Disc repair/reposition (1)	- 0.75 (95% CI: – 2.02 to 0.52) p = 0.123673 (2) - 0.47 (95% CI: – 1.00 to 0.06) p = 0.042119 (3) - 0.47 (95% CI: – 1.02 to 0.09) p = 0.050217 (4)	
			Arthrocentesis vs Disc repair/reposition (1)	- 0.83 (95% CI: – 2.35 to 0.70) p = 0.144515 (2) - 0.68 (95% CI: – 1.40 to 0.04) p = 0.032832 (3) - 0.68 (95% CI: – 1.43 to 0.07) p = 0.037370 (4)	

(1) Differences Between Subgroup Means

(2) Assumptions of 0% improvement in the absence of treatment

(3) Assumptions of 37.5% improvement in the absence of treatment

(4) Assumptions of 75% improvement in the absence of treatment

### The quality of the systematic reviews and overall quality of the evidence

The evaluation of the methodological quality of the included reviews for each instrument is shown in Tables 3. 4, 5, 6, 7, 8 and the overall quality results are shown in Table 9. The Kropmans et al review[27] met 23.5 ± 6.0% (ranging 18.2% to 30% 'yes' answers) and the Reston and Turkelson review[28] met 77.5 ± 12.8% (ranging 63.6 to 88.9% yes answers) of the methodological quality criteria (mean ± sd).

**Table 3:** AMSTAR component score results for Kropmans et al 1999[27]

*AMSTAR Question*	*YES*	*Can't Tell*	*No*
1. Was an 'a priori' design provided?	X		
2. Was there duplicate study selection and data extraction?	X		
3. Was a comprehensive literature search performed?			X
4. Was the status of publication (i.e. grey literature) used as an inclusion criterion?		X	
5. Was a list of studies (included and excluded) provided?		X	
6. Were the characteristics of the included studies provided?			X
7. Was the scientific quality of the included studies assessed and documented?			X
8. Was the scientific quality of the included studies used appropriately in formulating conclusions?			X
9. Were the methods used to combine the findings of studies appropriate?			X
10. Was the likelihood of publication bias assessed?			X
11. Was the conflict of interest stated?			X

**Table 4:** OQAQ component score results for Kropmans et al 1999[27]

*OQAQ Question*	*YES*	*Can't Tell*	*No*
1. Were the Search methods used to find evidence on the primary question(s) stated?			X
2. Was the search for evidence reasonably comprehensive?		X	
3. Were the criteria used for deciding which studies to include in the overview reported?	X		
4. Was bias in the selection of studies avoided?	X		
5. Were the criteria used for assessing the validity of the included studies reported?			X
6. Was the validity of all the studies referred to in the text assessed using appropriate criteria?			X
7. Were the methods used to combine the findings of the relevant (to reach a conclusion) reported?		X	
8. Were the findings of the relevant studies combined appropriately relative to the primary question of the overview?		X	
9. Were the conclusions made by the author(s) supported by the data and/or analysis reported in the overview?		X	

**Table 5:** CASP component score results for Kropmans et al 1999[27]

*CASP Question*	*YES*	*Can't Tell*	*No*
1. Did the review ask a clearly-focused question?	X		
2. Did the review include the right type of study?	X		
3. Did the reviewers try to identify all relevant studies?		X	
4. Did the reviewers assess the quality of the included studies?			X
5. If the results of the studies have been combined, was it reasonable to do so?			X
6. The main results are presented?		X	
7. Are these results precise?		X	
8. Can the results be applied to the local population?		X	
9. Were all important outcomes considered?	X		
10. Should policy or practice change as a result of the evidence contained in this review?		X	

**Table 6:** AMSTAR component score results for Reston and Turkelson 2003[28]

*AMSTAR Question*	*YES*	*Can't Tell*	*No*
1. Was an 'a priori' design provided?	X		
2. Was there duplicate study selection and data extraction?		X	
3. Was a comprehensive literature search performed?	X		
4. Was the status of publication (i.e. grey literature) used as an inclusion criterion?	X		
5. Was a list of studies (included and excluded) provided?		X	
6. Were the characteristics of the included studies provided?	X		
7. Was the scientific quality of the included studies assessed and documented?		X	
8. Was the scientific quality of the included studies used appropriately in formulating conclusions?		X	
9. Were the methods used to combine the findings of studies appropriate?	X		
10. Was the likelihood of publication bias assessed?	X		
11. Was the conflict of interest stated?	X		

**Table 7:** OQAQ component score results for Reston and Turkelson 2003[28]

*OQAQ Question*	*YES*	*Can't Tell*	*No*
1. Were the Search methods used to find evidence on the primary question(s) stated?	X		
2. Was the search for evidence reasonably comprehensive?	X		
3. Were the criteria used for deciding which studies to include in the overview reported?	X		
4. Was bias in the selection of studies avoided?	X		
5. Were the criteria used for assessing the validity of the included studies reported?	X		
6. Was the validity of all the studies referred to in the text assessed using appropriate criteria?			X
7. Were the methods used to combine the findings of the relevant (to reach a conclusion) reported?	X		
8. Were the findings of the relevant studies combined appropriately relative to the primary question of the overview?	X		
9. Were the conclusions made by the author(s) supported by the data and/or analysis reported in the overview?	X		

**Table 8:** CASP component score results for Reston and Turkelson 2003[28]

*CASP Question*	*YES*	*Can't Tell*	*No*
1. Did the review ask a clearly-focused question?	X		
2. Did the review include the right type of study?	X		
3. Did the reviewers try to identify all relevant studies?	X		
4. Did the reviewers assess the quality of the included studies?	X		
5. If the results of the studies have been combined, was it reasonable to do so?	X		
6. The main results are presented?	X		
7. Are these results precise?	X		
8. Can the results be applied to the local population?		X	
9. Were all important outcomes considered?	X		
10. Should policy or practice change as a result of the evidence contained in this review?		X	

**Table 9:** Quality of the Reviews

*Instrument*	*Kropmans et al 1999*[27]								*Reston and Turkelson 2003*[28]							
	YES		CAN'T TELL		NO		TOTAL		YES		CAN'T TELL		NO		TOTAL	

	n	%	n	%	n	%	n	%	n	%	n	%	n	%	n	%

AMSTAR	2	18.2	2	18.2	7	63.6	11	100.0	7	63.6	4	36.4	0	0.0	11	100.0
OQAQ	2	22.2	4	44.4	3	33.3	9	100.0	8	88.9	0	0.0	1	11.1	9	100.0
CASP	3	30.0	5	50.0	2	20.0	10	100.0	8	80.0	2	20.0	0	0.0	10	100.0

**Mean**	23.5								77.5							
**Standard Deviation**	6.0								12.8							

Table 10 summarizes the quality of evidence in each review. Data from 20 articles (4585 patients) were analyzed by Kropmans et al[27]. Data from 23 articles (1463 patients) were analyzed by Reston and Turkelson[28]. Only three (15%) articles in Kropmans et al[27] review were randomized clinical trials (high quality evidence), which represents 1.6% of the number of patients and six (30%) were Quasi-RCT (moderate evidence), representing 6% of the patients. In this case, methodological quality varied between RCTs in terms of how the sequence generation was done and the adequacy of allocation concealment. Conversely, eleven (55%) of the articles were non-systematic clinical observations (ie, case reports and case series), which were classified as very-low-quality evidence and represents 92.3% of the number of patients. The analysis of the included articles in Reston and Turkelson[28] showed that two (8.7%) were randomized clinical trials, which represents 7.7% of the number of patients, five (21.7%) were observational studies (low evidence), which represents 29% of the patients, and 16 (69.6%) were case series, which represents 63.3% of the number of patients.

**Table 10:** Quality of the Evidence

*Study Design*	Quality	*Kropmans et al 1999[27]*				*Reston and Turkelson 2003[28]*			
		Articles		Patients		Articles		Patients	

		n	%	n	%	n	%	n	%

RCT	High	3	15.0	74	1.6	2	8.7	112	7.7
Quasi-RCT	Moderate	6	30.0	277	6.0	0	0.0	0	0.0
Observational Study	Low	0	0.0	0	0.0	5	21.7	425	29.0
Any Other Evidence	Very Low	11	55.0	4234	92.3	16	69.6	926	63.3

**Total**		20	100.0	4585	100.0	23	100.0	1463	100.0

## Discussion

In this report, we sought to identify and evaluate the quality of systematic reviews comparing surgical and non-surgical care of TMJD. Our search strategy identified two reviews (one with and one without a meta-analysis). Kropmans et al[27] found no difference in clinical outcomes (e.g.: maximal mouth opening, pain, or function improvement) when comparing arthroscopic surgery, arthrocentesis, and physical therapy. Similarly, Reston and Turkelson[28] examining the efficacy of the surgical techniques arthrocentesis, arthroscopy, and disc repair/reposition, found no difference in clinical outcomes. Conceptually, both studies provide estimates of surgical vs. surgical and non surgical care, and both indicate that there are no significant differences between the interventions examined (Table 2). However, the methodological quality (from 0 to 100, with 100 being the best) of these two systematic reviews (Kropmans et al[27] = 23.5; Reston and Turkelson[28] = 77.5), and the underlying high quality evidence upon which they are based (from 0 to 100, with 100 being the best) (Kropmans et al[27] = 15; Reston and Turkelson[28] = 8.7), suggests that the interpretations of these authors may be over- (or under-) statements. This leads one to an interesting dilemma: How can one best care for patients if there are few high quality studies upon which to base this care?

The authors of the systematic reviews recognized some of these shortcomings. Reston and Turkelson[28] pointed out that their study is an explicit attempt to provide a conclusion when reliable primary data are not available and recognized that clinical and policy decisions must often be made in the absence of well-designed trials. They also discussed two main flaws. The first is that there are few studies that evaluated a specific treatment on specific TMJD sub-groups of patients; therefore the relative efficacy of each treatment could not be defined. The other is that the studies contained a wide range of definitions of success or improvement after treatment. Kropmans et al[27] reported that none of the reviewed scientific papers reported the measurement error of the procedures used (e.g.: standard error of measurement or 95% confidence interval).

Another set of issues is the classification of trials design. There are 8 articles common to both reviews, from which 3 match the same study design classification and 5 don't match. This could be explained by differences in the way that the included systematic reviews classified the study design of each article. A possible solution to this problem is the use of standard approaches to classify study designs, thus providing more consistency (e.g.:[29]). These approaches has been used successfully by other areas of dental care (e.g.: preventive dentistry[30,31]).

While the overall quality of the reviews is of some interest, the results of each measurement tool (Tables 3, 4, 5, 6, 7, 8) may offer additional insight into areas that could be improved. For example, the common missing elements in systematic reviews, in general, are: explicit search strategy; explicit articulation of exclusion criteria; absence of quality assessment of the underlying studies; and inappropriate aggregation of study results[32]. Thus, there are crucial elements in the conduct of a systematic review, without which the results may be questionable. From the analysis of Tables 3, 4, 5, 6, 7, 8 one can identify that each included systematic review showed different results for each instrument. This could be explained by the fact that each instrument has a question pattern to appraise methodological quality and address different key areas. For example, AMSTAR addresses to potential bias in systematic reviews (such as: funding source and conflict of interest) that OQAQ and CASP doesn't.

Ideally, systematic reviews should be the starting point for any search for information[33]. The results of the current study raise questions about the quality of the systematic reviews and clinical trials upon which TMJD care is based. Unfortunately, this is a long standing systematic problem. A systematic review of TMJD diagnosis[34], which was carried out more than 10 years ago, reported major to extensive methodologic flaws in 40% of the analyzed studies. Similarly, in examining the clinical literature, Bader and Ismail[18] identified 7 systematic reviews on diagnosis and non-surgical treatment of TMJD. For 6 of the reviews (85.7%) they identified a need for well designed controlled studies, the use of standardized diagnostic criteria and outcome measures. Finally a 2007 report from the United States Government Accountability Office[35] examined the Food and Drug Administration agency approval for the use of temporomandibular joint implants. They identified some of the same methodological problems: inadequate or inaccurate measurements, sample size and patient follow-up.

Thus, many of the previous results and this systematic review are congruent. That is, clinical scientists need to begin designing, implementing, and reporting clinical trials and systematic reviews that meet international standards[36]. In terms of international standards and chronic pain, there is the IMMPACT[37] recommendations, which suggest that chronic pain clinical trials should assess outcomes representing six core domains: (1) pain, (2) physical functioning, (3) emotional functioning, (4) participant ratings of improvement and satisfaction with treatment, (5) symptoms and adverse events, (6) participant disposition (e.g. adherence to the treatment regimen and reasons for premature withdrawal from the trial).

More specifically, for TMJD, a coalescence of symptom and outcome measurements could be profound. For example, one might consider measurements and reporting of pain as pioneered by the Oxford Pain Research Center[38]. This Pain Center, in generating or evaluating systematic reviews of acute pain, prefers validated visual analog scales (VAS) to report pain, specifically includes randomised, double-blind, single-dose studies in patients with moderate to severe pain, and looks for outcomes of 50% pain relief at 4–6 hours. The analog for TMJD could be VAS of symptoms pre-therapy and 3-months post-therapy. Post-therapy reporting would include both the number and percentage of patients with 50% pain relief for both the experimental and control group.

This would, however, necessitate having clinical scientists register their trials, select and implement interventions and comparisons in a standard, randomized, blinded fashion, and completely report these results using the CONSORT guideline (e.g.:[39]). Were this to occur it would substantially improve the evidence-base, reduce the variation in care and improve knowledge of patient outcomes. Moreover, there are also guidelines (e.g.: TREND[40]) to help researchers improve the transparency or clarity of non-randomized designs reports.

There are at least four limitations to our study. First, several instruments exist to assess the methodological quality of systematic reviews[41], but not all of them have been developed systematically or empirically validated and have achieved general acceptance[19]. Furthermore, since their development, considerable empirical research has accumulated about potential sources of bias in systematic reviews. For example, recent methodological research has highlighted the potential importance of publication language and publication bias in systematic reviews[19]. As an attempt to address the slightly varying perspectives of these instruments, we averaged the outcomes of each instruments. Moreover, the average of the group of does not differ radically from the assessment of any individual instrument. This suggests that the average is a reasonable estimate of the quality.

Second, our searches identified 211 studies, of which 22 (10.4%) were in excluded languages. Thus, while we attempted to capture the majority of the published literature, we clearly missed a significant portion of the literature. Third, we did not examine the reference lists of the identified articles, further increasing the probability of missed information. Forth, to extrapolate our results to the care of individual patients would be difficult. All patients vary with regard to their pathology and clinical characteristics and need a personalized approach to care. Although the overall outcomes of TMJ surgery in clinical trials may not be different than non-surgical care, TMJ surgery may still be the best option for specific patients and should be a treatment that should not be forgotten.

The most troubling aspect of our findings involves the ethics of trials that do not meet international standards of conduct, and the care of patients that is not based on high levels of evidence. The potential implications of this failing is clearest to understand in terms of the U.S. Supreme Court ruling in Daubert v. Merrell Dow[42]. In this case the Supreme Court applied the Federal Rules of Evidence[43] for causality of harm, based on the highest level of evidence. This ruling supplanted the common-law test of Frye v. United States, which based rulings on local practice customs. Thus one might imagine that legal suits could arise from the application of trial methodology or clinical practice that does not meet international standards.

## Conclusion

We identified two systematic reviews that compared surgical and non-surgical treatment of TMJD. Both studies indicated that there are no statistically significant differences between the effects of the identified treatments. However, both studies, while of different methodological quality, could only identify, and thus based their outcomes on studies of mixed quality. This raises 2 concerns. First, we are basing clinical care on studies of low quality and second there is a clear need for considerably more attention to clinical trial design, implementation and reporting, if one is to provide quality patient care.

## Competing interests

There are no competing interests. This work was part of a thesis, support by the Brazilian Council for Science and Technology (CNPq), submitted to the faculty of graduate studies in partial fulfilment of the requirement for the degree of PhD, Department of Oral and Maxillofacial Surgery, University of Pernambuco, Brazil.

## Authors' contributions

RN and BCEV conceived the study. RVBN and RN were responsible for the design of the study, searching, selection and data acquisition and analysis. All authors were involved in the drafting of the manuscript and gave approval of the final version.

## Pre-publication history

The pre-publication history for this paper can be accessed here:



## Supplementary Material

Additional file 1 **References that appeared to meet the definition of systematic review or meta-analysis.** Click here for file
