# Supplementary material for: The methodological quality of systematic reviews comparing temporomandibular joint disorder surgical and non-surgical treatment
Source: BMC Oral Health. 2008 Sep 26;8:27. doi: 10.1186/1472-6831-8-27 (PMC2576167; doi:10.1186/1472-6831-8-27)
Supplement: Additional file 1 — References that appeared to meet the definition of systematic review or meta-analysis. [file 1472-6831-8-27-S1.pdf]

1. Abrahamsson C, Ekberg E, Henrikson T, Bondemark L: **Alterations of temporomandibular disorders before and after orthognathic surgery: a systematic review 9**. *Angle Orthod* 2007, **77**(4):729-734.
2. Angiero F, Vinci R, Sidoni A, Stefani M: **Mesenchymal chondrosarcoma of the left coronoid process: report of a unique case with clinical, histopathologic, and immunohistochemical findings, and a review of the literature**. *QuintessenceInt* 2007, **38**(4):349-355.
3. Sembronio S, Albiero AM, Robiony M, Costa F, Toro C, Politi M: **Septic arthritis of the temporomandibular joint successfully treated with arthroscopic lysis and lavage: case report and review of the literature**. *Oral SurgOral MedOral PatholOral RadiolEndod* 2007, **103**(2):e1-e6.
4. Bu SS, Jin SL, Yin L: **Superolateral dislocation of the intact mandibular condyle into the temporal fossa: review of the literature and report of a case**. *Oral SurgOral MedOral PatholOral RadiolEndod* 2007, **103**(2):185-189.
5. Abramowicz S, Marshall CJ, Dolwick MF, Cohen D: **Vascular malformation of the temporomandibular joint: report of a case and review of the literature**. *Oral SurgOral MedOral PatholOral RadiolEndod* 2007, **103**(2):203-206.
6. Reyes Macias JF, Sanchez PM: **Synovial chondromatosis of the temporomandibular joint**. *MedOral PatolOral CirBucal* 2007, **12**(1):E26-E29.
7. Dolwick MF: **Temporomandibular joint surgery for internal derangement**. *Dent Clin North Am* 2007, **51**(1):195-viii.
8. Acar GO, Cansiz H, Guvenc MG, Mercan H, Dervisoglu S: **Synovial chondromatosis of the temporomandibular joint with skull base extension**. *J CraniofacSurg* 2007, **18**(1):241-243.
9. Ohura N, Ichioka S, Sudo T, Nakagawa M, Kumaido K, Nakatsuka T: **Dislocation of the bilateral mandibular condyle into the middle cranial fossa: review of the literature and clinical experience**. *J Oral MaxillofacSurg* 2006, **64**(7):1165-1172.
10. Malta J, Campolongo GD, Barros TEPd, Oliveira RPd: **Eletromiografia aplicada aos músculos da mastigação**. *Acta ortop bras* 2006, **14**(2):106-107.
11. van der Wal KG: **[Dissertations 25 years after the date 7. Temporomandibular joint ankylosis]**. *NedTijdschrTandheelkd* 2005, **112**(10):380-384.
12. Daniels JS, Ali I: **Post-traumatic bifid condyle associated with temporomandibular joint ankylosis: report of a case and review of the literature**. *Oral SurgOral MedOral PatholOral RadiolEndod* 2005, **99**(6):682-688.
13. Guven O: **Inappropriate treatments in temporomandibular joint chronic recurrent dislocation: a literature review presenting three particular cases**. *J CraniofacSurg* 2005, **16**(3):449-452.
14. Dimitroulis G: **The role of surgery in the management of disorders of the temporomandibular joint: a critical review of the literature. Part 2**. *Int J Oral MaxillofacSurg* 2005, **34**(3):231-237.

15. Dimitroulis G: **The role of surgery in the management of disorders of the Temporomandibular Joint: a critical review of the literature. Part 1**. *Int J Oral MaxillofacSurg* 2005, **34**(2):107-113.
16. Indresano AT, Casagrande A: **Temporomandibular joint disease: an update of surgical treatment**. *J CalifDent Assoc* 2004, **32**(10):845-848.
17. Reiter AM: **Symphysiotomy, symphysiectomy, and intermandibular arthrodesis in a cat with open-mouth jaw locking--case report and literature review**. *J VetDent* 2004, **21**(3):147-158.
18. Simon E, Chassagne JF, Dewachter P, Boisson-Bertrand D, Dumont T, Bussienne JE, Sellal S: **[Report on temporo-mandibular ankylosis. XXXIXe Congress of the French Society of Stomatology and Maxillofacial Surgery]**. *RevStomatolChir Maxillofac* 2004, **105**(2):71-124.
19. Martin-Granizo R, Caniego JL, de PM, Dominguez L: **Arteriovenous fistula after temporomandibular joint arthroscopy successfully treated with embolization**. *Int J Oral MaxillofacSurg* 2004, **33**(3):301-303.
20. Dzhorov A: **[Surgical methods for reconstruction of the temporomandibular joint and lower jaw in ankylosis and micrognathism]**. *Khirurgiia (Sofiia)* 2004, **60**(6):34-39.
21. Agurto P J, Mardones M M, Nunez B C: **Hiperplasia coronoidea**. *Rev otorrinolaringol cir cabeza cuello* 2004, **64**(1):32-38.
22. Herford AS, Boyne PJ: **Ankylosis of the jaw in a patient with fibrodysplasia ossificans progressiva**. *Oral SurgOral MedOral PatholOral RadiolEndod* 2003, **96**(6):680-684.
23. Detamore MS, Athanasiou KA: **Motivation, characterization, and strategy for tissue engineering the temporomandibular joint disc**. *Tissue Eng* 2003, **9**(6):1065-1087.
24. Wolford LM: **Concomitant temporomandibular joint and orthognathic surgery**. *J Oral MaxillofacSurg* 2003, **61**(10):1198-1204.
25. Mardinger O, Rosen D, Minkow B, Tulzinsky Z, Ophir D, Hirshberg A: **Temporomandibular joint involvement in malignant external otitis**. *Oral SurgOral MedOral PatholOral RadiolEndod* 2003, **96**(4):398-403.
26. Mercuri LG, Anspach WE, III: **Principles for the revision of total alloplastic TMJ prostheses**. *Int J Oral MaxillofacSurg* 2003, **32**(4):353-359.
27. DaSilva AF, Shaefer J, Keith DA: **The temporomandibular joint: clinical and surgical aspects**. *Neuroimaging Clin NAm* 2003, **13**(3):573-582.
28. Nitzan DW: **Rationale and indications for arthrocentesis of the temporomandibular joint**. *AlphaOmegan* 2003, **96**(2):57-63.
29. Hall HD: **Temporomandibular joint surgery meta-analysis; pain relief as an outcome measure**. *J Oral MaxillofacSurg* 2003, **61**(7):851.
30. Greene CS, Obrez A: **Mandibular repositioning in the treatment of temporomandibular disorder: critical analysis**. *AlphaOmegan* 2003, **96**(2):40-46.

31. Ugboko VI, Amole AO, Ibitoye B: **Applications of ultrasonography in oral and maxillofacial surgery: a review of the literature**. *AfrJ MedMedSci* 2003, **32**(2):119-129.
32. Reston JT, Turkelson CM: **Meta-analysis of surgical treatments for temporomandibular articular disorders: a reply to the discussants**. *J Oral MaxillofacSurg* 2003, **61**(6):737-738.
33. Reston JT, Turkelson CM: **Meta-analysis of surgical treatments for temporomandibular articular disorders**. *J Oral MaxillofacSurg* 2003, **61**(1):3-10.
34. Morales Trejo B: **Evaluacion y conceptos de artrocentesis**. *Rev ADM* 2003, **60**(1):25-28.
35. Lauberer L, Perez Astete O: **Artroscopia de la articulacion temporomandibular**. *Acta otorrinolaringol* 2003, **15**(1):7-12.
36. Bjordal JM, Couppe C, Chow RT, Tuner J, Ljunggren EA: **A systematic review of low level laser therapy with location-specific doses for pain from chronic joint disorders 69**. *AustJPhysiother* 2003, **49**(2):107-116.
37. Casteigt J: **[Surgery of asymmetry]**. *OrthodFr* 2002, **73**(3):317-352.
38. von Lindern JJ, Theuerkauf I, Niederhagen B, Berge S, Appel T, Reich RH: **Synovial chondromatosis of the temporomandibular joint: clinical, diagnostic, and histomorphologic findings**. *Oral SurgOral MedOral PatholOral RadiolEndod* 2002, **94**(1):31-38.
39. Kohjitani A, Miyawaki T, Kasuya K, Mishima K, Sugahara T, Shimada M: **Anesthetic management for advanced rheumatoid arthritis patients with acquired micrognathia undergoing temporomandibular joint replacement**. *J Oral MaxillofacSurg* 2002, **60**(5):559-566.
40. Mercado Montanez F: **Reemplazo condilar total como tratamiento de la anquilosis temporomandibular: reporte de dos casos bilaterales**. *Rev ADM* 2002, **59**(1):34-39.
41. McKenna SJ: **Discectomy for the treatment of internal derangements of the temporomandibular joint**. *J Oral MaxillofacSurg* 2001, **59**(9):1051-1056.
42. Dolwick MF: **Disc preservation surgery for the treatment of internal derangements of the temporomandibular joint**. *J Oral MaxillofacSurg* 2001, **59**(9):1047-1050.
43. White RD: **Arthroscopic lysis and lavage as the preferred treatment for internal derangement of the temporomandibular joint**. *J Oral MaxillofacSurg* 2001, **59**(3):313-316.
44. Indresano AT: **Surgical arthroscopy as the preferred treatment for internal derangements of the temporomandibular joint**. *J Oral MaxillofacSurg* 2001, **59**(3):308-312.
45. Semkin VA, Liashev IN: **[Use of total endoprosthesis of the temporomandibular joint: State of the art in the world]**. *Stomatologiia (Mosk)* 2001, **80**(4):69-72.

46. Lovato CH, De Freitas Oliveira Paranhos H, Pardini LC: **Protesis total: protocolo de evaluacion de la instalacion**. *Rev Asoc Odontol Argent* 2001, **89**(4):399-404.
47. Glowacki J: **Engineered cartilage, bone, joints, and menisci. Potential for temporomandibular joint reconstruction**. *Cells Tissues Organs* 2001, **169**(3):302-308.
48. Casablanca Ibanez W: **ATM y disfuncion**. *Gac odontol* 2001, **3**(2):35-38.
49. Lee JH, Kim YY, Seo BM, Baek SH, Choi JY, Choung PH, Kim MJ: **Extra-articular pigmented villonodular synovitis of the temporomandibular joint: case report and review of the literature**. *Int J Oral MaxillofacSurg* 2000, **29**(6):408-415.
50. Strauss RA: **Lasers in oral and maxillofacial surgery**. *Dent Clin North Am* 2000, **44**(4):851-873.
51. Quinn P: **Pain management in the multiply operated temporomandibular joint patient**. *J Oral MaxillofacSurg* 2000, **58**(10 Suppl 2):12-14.
52. Naeije M, Lobbezoo F, van Loon LA, Savalle WP, van der ZJ, Huddleston Slater JJ, van der Meulen MJ, Visscher CM: **[Treatment protocol for craniomandibular dysfunction 2. Treatment]**. *NedTijdschrTandheelkd* 2000, **107**(10):406-412.
53. Aoyama S, Kino K, Amagasa T, Kayano T, Ichinose S, Kimijima Y: **Differential diagnosis of calcium pyrophosphate dihydrate deposition of the temporomandibular joint**. *BrJ Oral MaxillofacSurg* 2000, **38**(5):550-553.
54. Hasson O, Levy Y, Nahlieli O: **[Arthrocentesis and lavage of the temporomandibular joint: treatment of closed lock]**. *Harefuah* 2000, **139**(5-6):226-229.
55. Reich RH: **[Conservative and surgical treatment possibilities in temporomandibular joint diseases]**. *Mund Kiefer Gesichtschir* 2000, **4 Suppl 1**:S392-S400.
56. Carroll TA, Smith K, Jakubowski J: **Extradural haematoma following temporomandibular joint arthrocentesis and lavage**. *BrJ Neurosurg* 2000, **14**(2):152-154.
57. Barkin S, Weinberg S: **Internal derangements of the temporomandibular joint: the role of arthroscopic surgery and arthrocentesis**. *J CanDent Assoc* 2000, **66**(4):199-203.
58. Nahlieli O, Lewkowicz A, Hasson O, Vered M: **Ganglion cyst of the temporomandibular joint: report of case and review of literature**. *J Oral MaxillofacSurg* 2000, **58**(2):216-219.
59. Mercuri LG: **The use of alloplastic prostheses for temporomandibular joint reconstruction**. *J Oral MaxillofacSurg* 2000, **58**(1):70-75.
60. MacIntosh RB: **The use of autogenous tissues for temporomandibular joint reconstruction**. *J Oral MaxillofacSurg* 2000, **58**(1):63-69.

61. Pereira GdS, Duarte JM, Vilela EM: **Avaliacao da sintomatologia ocular em pacientes com disfuncao temporomandibular**. *Arq bras oftalmol* 2000, **63**(4):263-267.
62. Maglione HO, Zavaleta Ld: **Dolor orofacial: Caracteristicas. Neurofisiologia: mecanismos moduladores. Su vinculacion con alteraciones del movimiento mandibular**. *Rev Circ Argent Odontol* 2000, **28**(188):37-49.
63. Goudot P, Jaquinet AR, Richter M: **Cysts of the temporomandibular joint. Report of two cases**. *Int J Oral MaxillofacSurg* 1999, **28**(5):338-340.
64. Goga D, Fassio E, Fetissof F, Jan M: **Chondroblastoma of the temporomandibular region**. *J Oral MaxillofacSurg* 1999, **57**(10):1270-1272.
65. Kropmans TJ, Dijkstra PU, Stegenga B, de Bont LG: **Therapeutic outcome assessment in permanent temporomandibular joint disc displacement**. *J Oral Rehabil* 1999, **26**(5):357-363.
66. Israel HA: **Part I: The use of arthroscopic surgery for treatment of temporomandibular joint disorders**. *J Oral MaxillofacSurg* 1999, **57**(5):579-582.
67. Frost DE, Kendell BD: **Part II: The use of arthrocentesis for treatment of temporomandibular joint disorders**. *J Oral MaxillofacSurg* 1999, **57**(5):583-587.
68. Stojadinovic S, Reinert S, Wildforster U, Jundt G: **Destruction of the glenoid joint fossa by a tenosynovial giant-cell tumour of the skull base: a case report**. *Int J Oral MaxillofacSurg* 1999, **28**(2):132-134.
69. Lee JJ, Worthington P: **Reconstruction of the temporomandibular joint using calvarial bone after a failed Teflon-Proplast implant**. *J Oral MaxillofacSurg* 1999, **57**(4):457-461.
70. Roychoudhury A, Parkash H, Trikha A: **Functional restoration by gap arthroplasty in temporomandibular joint ankylosis: a report of 50 cases**. *Oral SurgOral MedOral PatholOral RadiolEndod* 1999, **87**(2):166-169.
71. Mercuri LG: **Considering total temporomandibular joint replacement**. *Cranio* 1999, **17**(1):44-48.
72. Zanettini I, Zanettini UM: **Desordens temporomandibulares: estudo retrospectivo de 150 pacientes**. *Rev cient AMECS* 1999, **8**(1):9-15.
73. Kempers KG, Quinn PD, Silverstein K: **Surgical approaches to mandibular condylar fractures: a review**. *J CraniomaxillofacTrauma* 1999, **5**(4):25-30.
74. Hirschhaut M: **Desordenes temporomandibulares y dolor facial cronico**. *Rev venez ortod* 1999, **16**(1):480-486.
75. Baker GI: **Surgical considerations in the management of temporomandibular joint and masticatory muscle disorders**. *J OrofacPain* 1999, **13**(4):307-312.
76. Caminiti MF, Weinberg S: **Chronic mandibular dislocation: the role of non-surgical and surgical treatment**. *J CanDent Assoc* 1998, **64**(7):484-491.
77. Mercuri LG: **Alloplastic temporomandibular joint reconstruction**. *Oral SurgOral MedOral PatholOral RadiolEndod* 1998, **85**(6):631-637.

78. Yoda T: **Arthroscopic surgery and arthrocentesis of the temporomandibular joint--recent advances and long-term results**. *J MedDent Sci* 1997, **44**(4):71-74.
79. Wang D: **[Progresses in the study of stomatology in China, 1997]**. *Zhonghua YiXueZa Zhi* 1997, **77**(12):919-920.
80. Posnick JC: **Treacher Collins syndrome: perspectives in evaluation and treatment**. *J Oral MaxillofacSurg* 1997, **55**(10):1120-1133.
81. Sandler NA, Macmillan C, Buckley MJ, Barnes L: **Histologic and histochemical changes in failed auricular cartilage grafts used for a temporomandibular joint disc replacement: a report of three cases and review of the literature**. *J Oral MaxillofacSurg* 1997, **55**(9):1014-1019.
82. Richter M, Dulguerov P, Pittet B, Becker M: **Immediate function of temporomandibular joint after total resection and reconstruction**. *J CraniofacSurg* 1997, **8**(5):383-390.
83. Li KK, Ung F, McKenna MJ, Keith DA: **Combined middle cranial fossa and preauricular approach to the temporomandibular joint: report of a case**. *J Oral MaxillofacSurg* 1997, **55**(8):851-852.
84. Yang D, Han K: **[Clinical significance of temporomandibular joint disk displacement]**. *Zhonghua Kou QiangYiXueZa Zhi* 1997, **32**(3):183-185.
85. Tanaka K, Suzuki M, Nameki H, Sugiyama H: **Pigmented villonodular synovitis of the temporomandibular joint**. *Arch OtolaryngolHead Neck Surg* 1997, **123**(5):536-539.
86. Salgarelli A, Magnato R, Carminati R, Barbaglio A, Nocini PF: **[Myositis ossificans of the pterygoid and temporal muscles with metachronous contralateral involvement]**. *Minerva Stomatol* 1997, **46**(5):259-265.
87. Bradley PF: **A review of the use of the neodymium YAG laser in oral and maxillofacial surgery**. *BrJ Oral MaxillofacSurg* 1997, **35**(1):26-35.
88. Wolford LM: **Temporomandibular joint devices: treatment factors and outcomes**. *Oral SurgOral MedOral PatholOral RadiolEndod* 1997, **83**(1):143-149.
89. Milam SB: **Failed implants and multiple operations**. *Oral SurgOral MedOral PatholOral RadiolEndod* 1997, **83**(1):156-162.
90. Marbach JJ, Raphael KG: **Future directions in the treatment of chronic musculoskeletal facial pain: the role of evidence-based care**. *Oral SurgOral MedOral PatholOral RadiolEndod* 1997, **83**(1):170-176.
91. Giraud O, Lockhart R, Dichamp J, Capelle L, Kujas M, Dupuis HJ, Bertrand JC: **[Chondrosarcoma of the temporomandibular joint. Apropos of a case and review of the literature]**. *RevStomatolChir Maxillofac* 1997, **98**(1):2-6.
92. Fleury JE, Deboets D, Maffre M, Assaad C, Ferrey G: **[Critical review of the various treatments of temporomandibular joint pain-dysfunction syndrome. What will be the psychosomatic approach to these patients?]**. *RevStomatolChir Maxillofac* 1997, **98**(1):50-54.

93. Dolwick MF: **The role of temporomandibular joint surgery in the treatment of patients with internal derangement**. *Oral SurgOral MedOral PatholOral RadiolEndod* 1997, **83**(1):150-155.
94. Scarbrough FE, Wittenberg JM, Smith BR, Adcock DK: **Pulmonary edema following postoperative laryngospasm: case reports and review of the literature**. *AnesthProg* 1997, **44**(3):110-116.
95. Sandoval GP, Yepes A, Tamayo PA, Tarazona VJ, Tarazona CE, Tarazona RF: **Articulacion temporomandibular: sindromes dolorosos miofaciales**. *Acta otorrinolaringol cir cabeza cuello* 1997, **25**(1):35-40.
96. Micheli B: **[Temporomandibular joint disorders and surgery of the temporomandibular joint]**. *RevBelge MedDent* 1997, **52**(1):237-257.
97. Kendell BD, Frost DE: **Arthrocentesis**. *AtlasOral MaxillofacSurgClin North Am* 1996, **4**(2):1-14.
98. Donlon WC: **Associated bony procedures for preservation**. *AtlasOral MaxillofacSurgClin North Am* 1996, **4**(2):107-117.
99. de Bont LG, Dijkgraaf LC, Spijkervet FK: **[Arthroscopy, arthroscopic surgery and arthrocentesis of the temporomandibular joint]**. *NedTijdschrTandheelkd* 1996, **103**(7):258-262.
100. Karras SC, Wolford LM, Cottrell DA: **Concurrent osteochondroma of the mandibular condyle and ipsilateral cranial base resulting in temperomandibular joint ankylosis: report of a case and review of the literature**. *J Oral MaxillofacSurg* 1996, **54**(5):640-646.
101. Kryshtalskyj B, Weinberg S: **Surgical arthroscopy of the temporomandibular joint**. *OntDent* 1996, **73**(2):40-42.
102. Youssef RE, Roszkowski MJ, Richter KJ: **Pigmented villonodular synovitis of the temporomandibular joint**. *J Oral MaxillofacSurg* 1996, **54**(2):224-227.
103. Dimitroulis G, Dolwick MF: **Temporomandibular disorders. Part 3. Surgical treatment**. *AustDent J* 1996, **41**(1):16-20.
104. Vergara Munoz S: **Uso de la condilotomia modificada como alternativa de tratamiento para desplazamiento anterior del disco con reduccion y sintomatologia dolorosa de la ATM**. s.n; 1996.
105. Gola R, Carreau JP, De MG: **[Mandibular condyle hyperplasia. Therapeutic review]**. *RevStomatolChir Maxillofac* 1996, **97**(3):145-160.
106. Sanders B: **Management of internal derangements of the temporomandibular joint**. *SeminOrthod* 1995, **1**(4):244-257.
107. Stein JI: **TMJ arthrocentesis. A conservative surgical alternative**. *NYState Dent J* 1995, **61**(9):68-76.
108. Vezeau PJ, Fridrich KL, Vincent SD: **Osteochondroma of the mandibular condyle: literature review and report of two atypical cases**. *J Oral MaxillofacSurg* 1995, **53**(8):954-963.
109. Hensher R: **Temporomandibular joint replacement**. *BrJ HospMed* 1995, **53**(9):455-456.

110. Ohnishi M: **[Pathology and treatment of temporomandibular joint disorders--intra-articular injection therapy and arthroscopic surgery]**. *Nippon Seikeigeka Gakkai Zasshi* 1995, **69**(5):358-374.
111. Chuong R, Piper MA, Boland TJ: **Osteonecrosis of the mandibular condyle. Pathophysiology and core decompression**. *Oral SurgOral MedOral PatholOral RadiolEndod* 1995, **79**(5):539-545.
112. Gerard N, Hendler BH: **Laser arthroscopy of the temporomandibular joint**. *Compend Contin Educ Dent* 1995, **16**(4):350, 352-350, 354.
113. Calderazzi A, Eligi C, Guidetti F, Cambi L, Eligi B, Melchiorre D, Battolla L, Falaschi F: **[Synovial chondromatosis of the temporomandibular joint: an occasional finding in association with an arthrogenic cyst. A case report]**. *RadiolMed(Torino)* 1995, **89**(4):522-525.
114. Lopez LEE, Garzón DAC: **Efecto del factor de crescimento insulinico(IGF-1) sobre condrocitos de la articulacion temporomandibular de cerdos in vitro**. 1995.
115. Guerra C: **Metodos para la determinacion de la dimension vertical en pacientes totalmente edentulos**. s.n; 1995.
116. Gomez Sanchez German E PTA: **Estudo histológico descriptivo de la insercion del haz superior del musculo pterigoideo externo en fetos humanos**. 1995.
117. Bateman CAV: **Estudo discriptivo macro-microscopico y radiografico del condilo mandibular, articulacion costocondral externo esternal de la clavicula y metatarsiano em humanos**. 1995.
118. Aldegheri A, Blanc JL, Cheynet F, Chossegros C, Pauzie F: **[Bone lengthening: application to the mandible. Review of the literature]**. *RevStomatolChir Maxillofac* 1995, **96**(5):335-341.
119. Levin SN, Ryan DE: **Evolution of temporomandibular joint arthroscopy. "Current status"**. *WisDent Assoc J* 1994, **70**(6):36-37.
120. Bach DE, Waite PD, Adams RC: **Autologous TMJ disk replacement**. *J Am Dent Assoc* 1994, **125**(11):1504-1510, 1512.
121. Dolwick MF, Dimitroulis G: **Is there a role for temporomandibular joint surgery?** *BrJ Oral MaxillofacSurg* 1994, **32**(5):307-313.
122. Quinn JH: **Arthroscopic management of temporomandibular joint disc perforations and associated advanced chondromalacia by discoplasty and abrasion arthroplasty: preliminary results**. *J Oral MaxillofacSurg* 1994, **52**(8):800-806.
123. Pavel F, Machado L: **Contemporary oral and maxillofacial surgery**. *J CalifDent Assoc* 1994, **22**(4):35-36.
124. Israel HA: **Current concepts in the surgical management of temporomandibular joint disorders**. *J Oral MaxillofacSurg* 1994, **52**(3):289-294.
125. Sanchez Torres J, Toranzo Fernandez JM: **Patologia quirurgica de la articulacion temporomandibular**. *ADM* 1994, **51**(4):217-225.

126. Ruiz Valero CA, Gonzalez Bonilla C: **Modificacion del abordaje endaural para la articulaci≤n temporomandibular. Tecnica quirurgica y revision de literatura**. *Univ odontol* 1994, **13**(26):23-27.
127. Cartier S, Chikhani L, Favre-Dauvergne E, Bertrand JC, Guilbert F, Vaillant JM: **[Temporomandibular ankylosis: an analysis of hospital records and a review of the recent literature]**. *RevStomatolChir Maxillofac* 1994, **95**(2):160-163.
128. Calil Mathias A, Diaz Sarabia A, Saito T: **Dislocamiento del disco articular: evolucion del proceso, factores desencadenantes y consecuencias**. *Rev estomatol Hered* 1994, **4**(1/2):27-31.
129. McGuirt WF, Jr., Myers EN: **Ganglion of the temporomandibular joint presentation as a parotid mass**. *OtolaryngolHead Neck Surg* 1993, **109**(5):950-953.
130. Holmlund AB: **Surgery for TMJ internal derangement. Evaluation of treatment outcome and criteria for success**. *Int J Oral MaxillofacSurg* 1993, **22**(2):75-77.
131. Goss AN: **Toward an international consensus on temporomandibular joint surgery. Report of the Second International Consensus Meeting, April 1992, Buenos Aires, Argentina**. *Int J Oral MaxillofacSurg* 1993, **22**(2):78-81.
132. Chuong R, Piper MA: **Avascular necrosis of the mandibular condyle-pathogenesis and concepts of management**. *Oral SurgOral MedOral Pathol* 1993, **75**(4):428-432.
133. Buckley MJ, Merrill RG, Braun TW: **Surgical management of internal derangement of the temporomandibular joint**. *J Oral MaxillofacSurg* 1993, **51**(1 Suppl 1):20-27.
134. Mercier J, Adam P, Billet J, Cudia G: **[Chronic and neglected luxation of the temporomandibular joint]**. *RevStomatolChir Maxillofac* 1993, **94**(2):65-73.
135. Kent JN, Block MS, Halpern J, Fontenot MG: **Long-term results on VK partial and total temporomandibular joint systems**. *J LongTermEffMedImplants* 1993, **3**(1):29-40.
136. Garcia y Sanchez JM, Gomez Pedroso Balandrano A, Vargas Lopez D, Rengifo Lozano CA: **Tratamiento definitivo para el dolor de la articulacion temporomandibular**. *Pract odontol* 1993, **14**(5):11-18.
137. Contreras JR: **Perdida dentaria: su significacion en la etiologia de la disfuncion temporo mandibular**. s.n; 1993.
138. Shenaq SM: **Refinements in mandibular reconstruction**. *Clin PlastSurg* 1992, **19**(4):809-817.
139. Reich RH: **Temporomandibular joint surgery**. *CurrOpinDent* 1992, **2**:17-24.
140. Morgan DH: **Development of alloplastic materials for temporomandibular joint prosthesis: a historical perspective with clinical illustrations**. *Cranio* 1992, **10**(3):192-204.
141. Wilk BR, McCain JP: **Rehabilitation of the temporomandibular joint after arthroscopic surgery**. *Oral SurgOral MedOral Pathol* 1992, **73**(5):531-536.

142. Ketzler JT, Landers DF: **Management of a severed endotracheal tube during LeFort osteotomy**. *J Clin Anesth* 1992, **4**(2):144-146.
143. Heffez LB: **Arthroscopy broadens TMJ treatments**. *J Am Dent Assoc* 1992, **123**(3):107-102.
144. Eisig S, Dorfman HD, Cusamano RJ, Kantrowitz AB: **Pigmented villonodular synovitis of the temporomandibular joint. Case report and review of the literature**. *Oral SurgOral MedOral Pathol* 1992, **73**(3):328-333.
145. Moses JJ, Lo H: **The treatment of internal derangement of the temporomandibular joint--an arthroscopic approach**. *Oral SurgOral Diagn* 1992, **3**:5-11.
146. Morales H: **Disfuncion de la articulacion temporo mandibular**. *Odontol dia* 1992, **9**(4):12-18.
147. Fazari M, Kryshtalskyj B: **Diseases of the temporomandibular joint: surgical management**. *Univ TorDent J* 1992, **5**(2):7-11.
148. Dahl M, Sindet-Pedersen S, Jensen J, Cosentino SJ: **Arthroscopy of the human temporomandibular joint**. *Oral SurgOral Diagn* 1992, **3**:19-25.
149. Hoffman DR: **Surgical orthodontics**. *CurrOpinDent* 1991, **1**(5):645-651.
150. Keith DA: **Surgical treatment for temporomandibular joint problems**. *CurrOpinDent* 1991, **1**(4):503-506.
151. Upton LG, Sullivan SM: **The treatment of temporomandibular joint internal derangements using a modified open condylotomy: a preliminary report**. *J Oral MaxillofacSurg* 1991, **49**(6):578-583.
152. Hoffman D, Moses J, Topper D: **Temporomandibular joint surgery**. *Dent Clin North Am* 1991, **35**(1):89-107.
153. Tarro AW: **The treatment of TMJ disorders: a current update**. *J Mass Dent Soc* 1991, **40**(3):125-129.
154. Lander Hoffmann A, Espinoza M: **Reconstruccion de la articulacion temporomandibular con injertos costocondrales**. *Venezuela odontol* 1991, **56**(2):19-23.
155. Gonzalez Blanco O: **Estudio doble-ciego para establecer una correlacion entre los datos clinicos y de laboratorio sobre los sonidos de la articulacion temporomandibular**. s.n; 1991.
156. Cheynet F, Waller PY, Semeria E, Chossegros C, Gola R: **[Use of a posterior temporal muscle flap in surgery of the temporomandibular joint]**. *RevStomatolChir Maxillofac* 1991, **92**(2):84-91.
157. Arismendi E JA, Restrepo Echevarria A, Cortes C N: **Dimension vertical: una revision**. *Rev Fac Odontol Univ Antioquia* 1991, **3**(1):35-44.
158. Wilson AW, Brown JS, Ord RA: **Psoriatic arthropathy of the temporomandibular joint**. *Oral SurgOral MedOral Pathol* 1990, **70**(5):555-558.
159. Dahl M, Sindet-Pedersen S, Jensen J, Westermark A: **[Arthroscopy and arthroscopic surgery of the temporomandibular joint]**. *Tandlaegebladet* 1990, **94**(16):648-654.

160. Poker ID, Hopper C: **Surgery for temporomandibular joint pain**. *Dent Update* 1990, **17**(7):291-297.
161. Hartog JM, Slavin AB, Kline SN: **Reconstruction of the temporomandibular joint with cryopreserved cartilage and freeze-dried dura: a preliminary report**. *J Oral MaxillofacSurg* 1990, **48**(9):919-925.
162. Gundlach KK: **Long-term results following surgical treatment of internal derangement of the temporomandibular joint**. *J CraniomaxillofacSurg* 1990, **18**(5):206-209.
163. Sacks H, Zelig D, Schabes G: **Recurrent temporomandibular joint subluxation and facial ecchymosis leading to diagnosis of Ehlers-Danlos syndrome: report of surgical management and review of the literature**. *J Oral MaxillofacSurg* 1990, **48**(6):641-647.
164. Trujillo Fandino JJ, Gonzalez TG, Santos T: **[Anterior disinsertion of the external pterygoid muscle by the oral approach as a treatment for chronic luxation]**. *PractOdontol* 1990, **11**(4):19-15.
165. Szabo G, Barabas J, Matrai J, Gyorgy J, Miklos L: **Application of compact aluminum oxide ceramic implants in maxillofacial surgery**. *J Oral MaxillofacSurg* 1990, **48**(4):354-361.
166. Totsuka Y, Fukuda H, Iizuka T, Shindoh M, Amemiya A: **Osteochondroma of the coronoid process of the mandible. Report of a case showing histological evidence of neoplasia**. *J CraniomaxillofacSurg* 1990, **18**(1):27-32.
167. Gundlach KK: **[Imaging procedures as principle aids in functional surgical therapy of the temporomandibular joint]**. *FortschrKiefer Gesichtschir* 1990, **35**:158-161.
168. Duvoisin B, Klaus E, Schnyder P, Jacques B: **Temporomandibular joint arthrography: normal anatomy and technique of examination**. *Radiol bras* 1990, **23**(1):1-4.
169. Buttram JR, Farole A: **Arthroscopy of the temporomandibular joint**. *Compendium* 1989, **10**(12):652, 654-652, 656.
170. Haug RH, Picard U, Matejczyk MB, Indresano AT: **The infected prosthetic total temporomandibular joint replacement: report of two cases**. *J Oral MaxillofacSurg* 1989, **47**(11):1210-1214.
171. Zins JE, Smith JD, James DR: **Surgical correction of temporomandibular joint ankylosis**. *Clin PlastSurg* 1989, **16**(4):725-732.
172. Greenberg SA, Jacobs JS, Bessette RW: **Temporomandibular joint dysfunction: evaluation and treatment**. *Clin PlastSurg* 1989, **16**(4):707-724.
173. McCain JP, de la RH: **Arthroscopic observation and treatment of synovial chondromatosis of the temporomandibular joint. Report of a case and review of the literature**. *Int J Oral MaxillofacSurg* 1989, **18**(4):233-236.
174. Sebastian MH, Moffett BC: **The effects of continuous passive motion on the temporomandibular joint after surgery. Part II. Appliance improvement, normal subject evaluation, pilot clinical trial**. *Oral SurgOral MedOral Pathol* 1989, **67**(6):644-653.

175. Poremba EP, Moffett BC: **The effects of continuous passive motion on the temporomandibular joint after surgery. Part I. Appliance design and fabrication**. *Oral SurgOral MedOral Pathol* 1989, **67**(5):490-498.
176. Losapio PL, Amaddeo P: **[A case of true congenital temporo-mandibular ankylosis]**. *Minerva Stomatol* 1989, **38**(5):505-508.
177. Markowitz NR, Allan PG, Duffy MT: **Reconstruction of the mandibular condyle using ramus osteotomies: a preliminary report**. *J Oral MaxillofacSurg* 1989, **47**(4):367-377.
178. Lessin ME, Gross PD: **Temporomandibular joint disorders: discussion of a rational approach to surgery and a retrospective analysis of surgeries performed**. *Oral SurgOral MedOral Pathol* 1989, **67**(4):374-378.
179. Greene MW, Hackney FL, Van Sickels JE: **Arthroscopy of the temporomandibular joint: an anatomic perspective**. *J Oral MaxillofacSurg* 1989, **47**(4):386-389.
180. Hasso AN, Christiansen EL, Alder ME: **The temporomandibular joint**. *RadiolClin North Am* 1989, **27**(2):301-314.
181. Luz JGdC, Affonso MMV, Jorge Wa: **Complicacoes das fraturas mandibulares**. *RGO* 1989, **37**(4):274-278.
182. Meyer RA: **The autogenous dermal graft in temporomandibular joint disc surgery**. *J Oral MaxillofacSurg* 1988, **46**(11):948-954.
183. Ioannides C, Freihofer HP: **Replacement of the damaged interarticular disc of the TMJ**. *J CraniomaxillofacSurg* 1988, **16**(6):273-278.
184. Behrman SJ, Behrman DA: **Oral surgeons' considerations in surgical orthodontic treatment**. *Dent Clin North Am* 1988, **32**(3):481-507.
185. Kryshtalskyj B, Weinberg S: **Surgical correction of internal derangements of the TMJ. The state of the art. Part One**. *Oral Health* 1988, **78**(5):19-16.
186. Kryshtalaskyj B, Weinberg S: **Surgical correction of internal derangements of the TMJ. The state of the art. Part Two**. *Oral Health* 1988, **78**(5):27-26.
187. Copeland M, Douglas B: **Ganglions of the temporomandibular joint: case report and review of literature**. *PlastReconstrSurg* 1988, **81**(5):775-776.
188. Quinn PD, Wedell D: **Complications from intraoral vertical subsigmoid osteotomy: review of literature and report of two cases**. *Int J AdultOrthodonOrthognathSurg* 1988, **3**(4):189-196.
189. Gallego Duque DHM, Claudia: **Sindrome de Disfuncion Meniscal intra-articular de la Articulacion Temporomandibular: con el Complemento de un Caso Clínico**. Fundacion Universitaria San Martin; 1988.
190. Cascone P: **[Surgical therapy of anterior luxation of the meniscus. 2]**. *Dent Cadmos* 1987, **55**(12):9, 11-19, 23.
191. Leopard PJ: **Surgery of the non-ankylosed temporomandibular joint**. *BrJ Oral MaxillofacSurg* 1987, **25**(2):138-148.
192. Harris M: **Medical versus surgical management of temporomandibular joint pain and dysfunction**. *BrJ Oral MaxillofacSurg* 1987, **25**(2):113-120.

193. Politis C, Erbe M, Bossuyt M, Fosson E: **[The role of costochondral grafts in the treatment of ankylosis of the maxillary joint]**. *RevBelge MedDent* 1987, **42**(6):172-178.
194. Given JW, Sanders B: **Surgical management of longstanding temporomandibular joint ankylosis and resultant skeletal deformity**. *J CraniomandibDisord* 1987, **1**(2):127-135.
195. Carrillo de Vetencourt AE: **Enfermedades sistemicas que inciden en la articulacion trempromandibular**. In *Enfermedades sistemicas que inciden en la articulacion trempromandibular*. Edited by Carrillo de Vetencourt AE. s.l: s.n; 1987:166.
196. Solberg WK: **Temporomandibular disorders: management of internal derangement**. *BrDent J* 1986, **160**(11):379-385.
197. Ash MM: **Current concepts in the aetiology, diagnosis and treatment of TMJ and muscle dysfunction**. *J Oral Rehabil* 1986, **13**(1):1-20.
198. Kent JN, Zide MF: **Wound healing: bone and biomaterials**. *OtolaryngolClin North Am* 1984, **17**(2):273-319.
199. Sisk AL: **Surgical treatment of chronic orofacial pain**. *AnesthProg* 1983, **30**(6):180-186.
200. Carlsson GE, Kopp S, Lindstrom J, Lundqvist S: **Surgical treatment of temporomandibular joint disorders. A review**. *SwedDent J* 1981, **5**(2):41-54.
201. Lebowitz MS, Hall MB, Laskin JL, Lucas WJ: **Oral and maxillofacial surgery: a review of the literature**. *FlaDent J* 1980, **51**(3):20-26.
202. Lasco GE, de Mello JB: **[Painful dysfunction of temporomandibular joint. Technic of conservative meniscus athroplasty. Comparative study with the meniscectomy]**. *Ars CurandiOdontol* 1979, **6**(4):48-54.
203. Morgan LR, Thompson CW: **Mandibular reconstruction. Current state of the art**. *Clin PlastSurg* 1975, **2**(4):561-576.
204. Miller GA, Page HL, Jr., Griffith CR: **Temporomandibular joint ankylosis: review of the literature and report of two cases of bilateral involvement**. *J Oral Surg* 1975, **33**(10):792-803.
205. Wallace L, Stern M: **Bilateral ankylosis of the mandible in the open position. Review of the literature and report of a case**. *Oral SurgOral MedOral Pathol* 1974, **37**(2):179.
206. Poswillo D: **Surgery of the temporomandibular joint**. *Oral Sci Rev* 1974, **6**(0):87-118.
207. Carlsson GE, Oberg T: **Remodelling of the temporomandibular joints**. *Oral Sci Rev* 1974, **6**(0):53-86.
208. Paul KK: **Surgery of the temporomandibular joint--a review**. *J Indian Dent Assoc* 1973, **45**(9):243-252.
209. Morgan DH: **Temporomandibular joint surgery. Correction of pain, tinnitus, and vertigo**. *Dent RadiogrPhotogr* 1973, **46**(2):27-39.
210. Silagi JL, Schow CE, Jr.: **Temporomandibular joint arthroplasty: review of literature and report of case**. *J Oral Surg* 1970, **28**(12):920-926.

211. Bell WE: **Recent concepts in the management of temporomandibular joint dysfunctions**. *J Oral Surg* 1970, **28**(8):596-599.
